# Journey toward a Global Understanding of Recombination in Halide Perovskites for Photovoltaic Applications

**DOI:** 10.1021/acsenergylett.6c00457

**Published:** 2026-05-12

**Authors:** Samuel D. Stranks, Thomas Kirchartz

**Affiliations:** † Department of Chemical Engineering and Biotechnology, 2152 University of Cambridge, Philippa Fawcett Drive, Cambridge, CB3 0AS, United Kingdom; ‡ Cavendish Laboratory, 2152 University of Cambridge, JJ Thomson Avenue, Cambridge, CB3 0US, United Kingdom; § IMD-3 Photovoltaics, Forschungszentrum Jülich, 52425, Jülich, Germany; ∥ Faculty of Engineering and CENIDE, University of Duisburg-Essen, Carl-Benz-Str. 199, 47057, Duisburg, Germany

## Abstract

Despite rapid progress in halide perovskite solar cells, a comprehensive understanding of recombination processes and agreement about the most appropriate models to explain recombination and interpret experimental data are still lacking yet will be critical to further performance and stability improvements. Here, we analyze the evolution of recombination models in halide perovskites, highlighting how the background of many researchers in dye-sensitized and organic photovoltaics triggered an early emphasis on the inclusion of excitons followed by a phase of simplifying the mathematical description until experiment and theory were clearly becoming inconsistent with each other. Recent years have seen the trend of going back to the original models for classical semiconductors. These approaches are more frequently combined with sophisticated approaches for fitting and quantifying confidence in data analysis that make increasing use of machine learning algorithms. We outline the need for global recombination models and the challenges and opportunities in attaining this.

## Graphical abstract

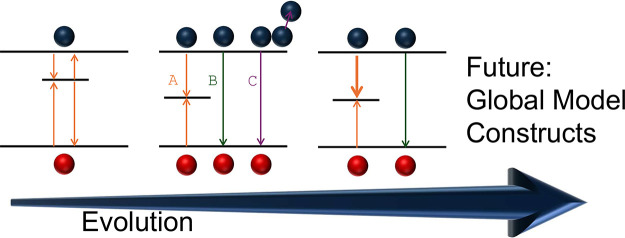

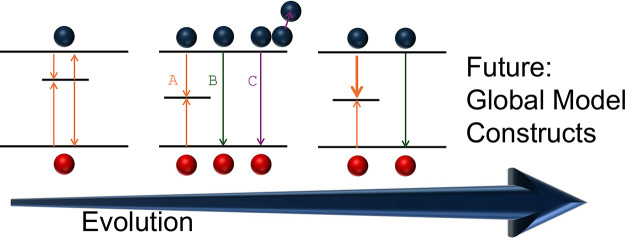

Halide perovskites are emerging semiconductors that are showing enormous promise in next-generation solar cells,^1^ lighting technologies^2^ and radiation detectors.^3^ These materials burst onto the energy materials scene in 2012 when the first perovskite solar cells beyond 10% efficiency were reported,^4,5^ spurring multiple new lines of research that today are actively worked on by thousands of groups worldwide, along with a growing industry.^6,7^ To direct efforts to maximize performance in semiconducting applications, such as solar cells, one needs to understand the fate of energized charge carriers. For example, key questions must be understood such as how efficiently carriers are generated, how efficiently they are collected at electrodes, and how they lose their energy through recombination events with carriers, contacts or defect states. These insights best inform how and where power losses occur in the device and therefore dictate where tangible improvements can be made to push efficiencies higher. The halide perovskite solar cell field has progressed so rapidly that device performance improvements have often outpaced genuine understanding of recombination. Nevertheless, pushing devices to their absolute efficiency limits or rethinking device structures will require a fundamental, global understanding that is currently lacking. Such an understanding will best enable applications such as multijunction solar cells and areas including radiation detectors, lighting, and quantum applications.

In addition, the highly interdisciplinary nature of the field has brought in a diversity of researcher backgrounds from different fields (arguably one of the reasons the field has been popularized and progressed so quickly) bringing their own preassumptions, but this has also led in some areas to legacy thinking that has clouded general understanding and best practice approach to optimization.

Here, we reflect on the evolution of the understanding of recombination in halide perovskites. We provide historical context into early insights and assumptions that have guided today’s understanding, and how some key misconceptions have been, or are being, adapted, expanded, or modified. We highlight how recombination models have evolved over time in both complexity and features. These developments have been in parallel to improvements in semiconductor quality and tools able to probe the processes. We make the case for a global recombination model construction able to describe all observations when appropriately adapted for the sample at hand, and outline the challenges in achieving this. Realizing such a lofty goal would allow a rational strategy to optimize device performance and stability across a range of device platforms underpinned by fundamental understanding, ensuring characterization tools can be used by a wide diversity of researchers to their maximum effect.

## Excitons, Luminescence Quenching and Diffusion

In the first years since the first high efficiency reports, a large fraction of the burgeoning perovskite solar cell community comprised those working on other emerging photovoltaic materials, primarily dye-sensitized solar cells (DSSCs) and organic photovoltaics (OPV). One common feature of DSSCs and OPVs is that the primary photoexcited species are excitons – strongly bound electrons and holes. In such configurations, devices must be designed to dissociate these excitons to give access to free electrons and holes that can be collected at opposite electrodes. Typical approaches include introduction of intermixed bulk heterojunctions or interfacing of dyes directly with collecting electrodes such as by adsorbing them on mesoporous metal oxide scaffolds. Given this exciton-focused community, the starting point for perovskite PV absorber materials was to also assume that excitons were the dominant photoexcited species. This assumption was seemingly validated by reports of magneto-optic measurements in earlier years^8,9^ interpreted in such a way that exciton binding energies were >50 meV and therefore significant. This contributed to early recombination models being complicated by excitons.^10^ Furthermore, it led to the fallacy that a quench of photoluminescence (PL) in the presence of a contact at open-circuit is a desired result for optimization^11,12^ – such a quench is the signature of necessary exciton dissociation in OPV systems,^13^ but this also leads to a penalty in open-circuit voltage in PV devices^14^ and should be avoided when dissociation is unnecessary.^15^ Indeed, the ideal solar cell should have maximized PL quantum efficiency (where all recombination is radiative) with no quench at open circuit, and minimum PL quantum efficiency and therefore maximum quench at short circuit. This can experimentally be accessed by voltage-dependent photoluminescence measurements that can of course only be performed on complete cells.^16,17^ The logical appeal to be able to perform measurements on samples without contacts (e.g., absorber plus electron or hole transport layer) has triggered various investigations into the usefulness of optical experiments on bilayers (i.e., half cells).^18–21^ Recently Gries et al. have shown^22^ that a combination of methods, including steady state PL, transient PL and transient surface photovoltage, can be used to infer the major loss mechanisms at interfaces with the transport layers. Here, the idea is that high steady-state PL is always a good sign for the open-circuit voltage, while the opposite is also true. In time-resolved PL (tr-PL), the situation is however slightly different. Here, an initial fast decay of a bilayer sample may be a good sign as it can indicate that, for instance, photogenerated electrons are quickly transferred from the perovskite layer to the initially empty electron transport layer until an equilibrium is created after which recombination of both populations of electrons with the holes in the perovskite will dominate the decay. However, as an initial fast decay may also be a sign of faster recombination, the combination of transient and steady-state PL^22,23^ might prove to become a useful combination of (often readily available) tools to arrive at correct conclusions about losses at interfaces.

Nevertheless, subsequent work made it clear that excitons are not predominant in those halide perovskite compositions that were most frequently used for photovoltaic applications. Key concepts that brought this to light include the fact that planar heterojunction solar cells, in which absorber films are simply sandwiched between collecting electron and hole layers, function efficiently,^4,24^ in turn consistent with reported electron and hole diffusion lengths being sufficiently long to allow suitable charge collection^25,26^ – both findings would be inconsistent with a strongly bound excitonic picture. Furthermore, further magneto-optic measurements in 2015 utilizing higher quality films than earlier reports,^27,28^ and the use of much higher magnetic fields to allow more robust spectroscopic fitting, showed that the exciton binding energy in such PV absorber materials is actually only a few millielectronvolts at room temperature. These results firmly established free electrons and holes in the absorber materials. Note that this conclusion is not generalizable to all halide perovskite materials as especially higher band gap^29^ or lower dimensional perovskite-inspired materials do show excitonic features.^30–32^

The perovskite PV materials are often described as having “long” charge-carrier diffusion lengths, in part based off the first reports of micron-long diffusion lengths^25^ and subsequent work on further improved samples over the years revealing even tens of micrometres.^33^ The concept of “long” is true when the starting point is the comparison to materials such as OPV absorbers, in which diffusion lengths are typically tens of nanometers and complicated bulk heterojunctions are required to ensure sufficient numbers of excitons hit electrodes and dissociate. However, when compared to other established semiconductors such as silicon in which diffusion lengths can be hundreds of micrometers to centimeters,^34^ this is not so long. Related to this, mobilities in halide perovskites remain only modest, ∼1–50 cm^2^/(Vs),^35^ orders of magnitude lower than for example GaAs.^36^ Nevertheless, the diffusion length can be considered “sufficiently long”^37^ for PV purposes when viewed in comparison to the absorption depth of photons from the solar spectrum: the typical absorber thicknesses are 500 nm, with an absorption depth of ∼50–200 nm, and diffusion lengths are at least many multiples of this, ensuring most carriers can be collected in the highest efficiency devices.^38^ The more salient point is that these materials can be processed quite crudely yet have a unique combination of suitably high absorption coefficient,^39^ low doping densities^40^ and only a moderately severe impact of traps. This combination allows lifetimes to remain long which, when combined with moderate mobilities, yields the suitably long diffusion properties.

## Deep Defects

One of the features of halide perovskites that became obvious early in the development of perovskite solar cells were the respectable open-circuit voltages (in comparison to the band gap) that could be achieved without excessive modifications in the device structure relative to the solid-state DSSC structure with a bulk perovskite layer. These open-circuit voltages were accompanied by initially modest^41,42^ but soon fairly high luminescence quantum efficiencies^43,44^ for the standards of a polycrystalline thin-film solar cell. The lower-than-unity luminescence quantum efficiencies were a clear indication of trap-assisted recombination (as one would expect), but it was still surprising that the problem was only moderately severe given the low maturity of the technology. An explanation that was brought forward was based on initial density functional theory calculations^45,46^ and suggested that intrinsic defects would likely be mostly shallow or even inside the bands with little to none of the defects lying deep within the band gap. As a shallow defect close to the conduction band is mostly empty (detrapping is fast relative to trapping), it does not contribute as much to recombination as compared to a deeper defect with similar characteristics. Furthermore, nonradiative transitions from band to defect (or vice versa) were traditionally expected to scale strongly with the energetic distance in units of the phonon energy.^47^ This would make an efficient transition of a shallow defect close to the conduction (valence) band to the valence (conduction) band orders of magnitude less likely than two transitions from either band edge to a midgap defect. Thus, the combination of mostly shallow defects, combined with particularly low phonon energies, seemed to provide a compelling argument for the simultaneous existence of a non-negligible concentration of defects and decently high luminescence quantum efficiencies. This phenomenon was then termed defect tolerance^48,49^ and the quest to precisely pinpoint its origin was especially prioritized by the community working on absorber material discovery. The trouble with the defect tolerance paradigm was that some of the main foundations of the theory turned out to be questionable. The initial DFT study^46^ lacked spin orbit coupling and was later replaced by others^50^ that showed that some of the intrinsic defects should be deep. The argument that shallow defects should be (nearly) unable to interact with the more distant band was based on the calculation of multiphonon recombination rates within the harmonic oscillator model^51^ which turned out to be particularly far off relative to more realistic calculations^52,53^ in the particularly anharmonic^54^ halide perovskites. Finally, most of the calculations of defect densities in thin films were found to be artifacts of the measurement method.^55,56^ Thus, the case of understanding the relatively low but still efficiency limiting amounts of defect-assisted recombination in halide perovskites is still far from settled.^57^ A likely scenario is that the initial ideas were neither entirely wrong nor the complete story.^57^ Thus, for halide perovskites the community continues to enjoy the perks of having to worry less about recombination than most other solar cell communities. However, the phenomenon is not sufficiently well understood to be able to find other materials with similarly low nonradiative recombination but e.g. higher stability or using less toxic elements.

## ABC Model

To mathematically analyze and describe recombination, the halide perovskite community had to use and apply recombination models that provided a good compromise between applicability and accuracy. Initial attempts to develop a recombination model were including a variety of different phenomena, including excitons, defects, photodoping^10^ and photon recycling.^58^ However, the first temporary point of convergence for the community^59,60^ became the fairly simple ABC model, in which each parameter (*A*, *B*, *C* or sometimes called *k*_1_, *k*_2_, *k*_3_) was typically associated with a single recombination mechanism (Shockley-Read-Hall [SRH], radiative, Auger-Meitner, respectively). A key benefit of the model is its simplicity and the ability to directly discriminate between the mechanisms based on their dependence on carrier density. Thus, every experimental method sensitive to recombination and some assay of carrier density would provide a way to distinguish the different mechanisms. As nearly all experimental studies of recombination both in steady state (e.g., luminescence quantum efficiencies vs injection conditions) or time domain (transient photoluminescence or transient absorption) would be able to notice recombination terms that scaled linearly and those that scaled quadratically (or nonlinearly) with carrier density, it was immediately clear from experimental data that at least a linear and quadratic recombination term were needed to describe recombination in halide perovskites. The cubic (Auger-Meitner, *C*) term was visible at high injection conditions only reached in a smaller subset of experiments.^44,59^

An essential feature of the ABC model is that it only considers one species (electrons). Within the context of a semiconducting device, this implies assuming that the electron and hole densities are always identical as is the case in an intrinsic semiconductor with negligible densities of charged defects. Evidence for the low doping density of halide perovskites originated initially from, e.g., the shape of cross-sectional electron-beam-induced current measurements.^61^ Later, Hall measurements^62^ as well as the absence of any features of device-relevant doping levels in a range of other experiments (fluence-dependent tr-PL, charge-extraction with linearly increased voltage, and Mott–Schottky) provided strong support to treat most lead-halide perovskites with band gaps relevant for photovoltaics as essentially intrinsic.^40^ This does not imply that the Fermi-level of a perovskite film in the dark is close to midgap, but that once the semiconductor is contacted and/or illuminated the position of the Fermi-level in the dark is of no relevance anymore for the functionality of the device. Given that photoexcitation always leads to the creation of equal densities of electrons and holes, the simplification of using only one species of charge carrier may have seemed sensible.

Within the ABC model, trap-assisted recombination was typically only associated with the parameter *A*. When comparing this assumption with the logic of the SRH recombination model, this was equivalent to assuming that only deep traps (close to midgap) are involved in recombination. The shallow traps that should be abundant according to DFT calculations could neither be involved in recombination nor could they start trapping a significant density of electrons or holes as this would have led to an asymmetry in free electron and hole densities. Several publications^63–65^ also noted that the quadratic term *B* seemed not always to be representing purely radiative processes but the fact that shallow traps can produce nonradiative recombination that can appear bimolecular (quadratic in free carrier density) was not discussed in the community until quite recently.^66^ Such a component can contribute to significant nonradiative losses even in high-performance devices.^67^

Evidence inconsistent with the ABC model was slowly building up over time until it was difficult to ignore the problem any longer. Even though many relevant lead-halide perovskite compositions behaved like undoped semiconductors in the dark, once sufficiently illuminated there were features emerging^68,69^ that were explained most easily with asymmetric trapping and subsequent differences in free electron and hole densities. These features included the power-law decays in transient PL combined with PL quantum efficiencies from steady-state PL. As power law decays result from any recombination mechanism that is quadratic in electron density in an intrinsic semiconductor, a power law could also be consistent with purely radiative recombination. However, this scenario can be ruled out for nearly all samples that show power laws as their steady-state PL quantum efficiency is too far away from unity to be consistent with radiative recombination dominating the decay. Further, many samples showed ideality factors of 1.5 in steady-state PL on films,^70,71^ which are impossible to explain other than by the presence of a significant density of charged traps. The final observation was that tr-PL and transient absorption data performed on the same sample^68^ could only be explained by asymmetries between electron and hole densities that only existed under illumination but not in the dark. This concept of photodoping was already included in early 2014 recombination models^10^ but had then disappeared again from the models during the time when ABC was most popular. Furthermore, more complicated variants of tr-PL measurements were developed e.g. by Scheblykin and co-workers.^72,73^ In these measurements, parameters such as repetition rate and laser power were varied over orders of magnitude, which led to data sets with a huge discriminatory power between models. This discriminatory power was so significant that, in some of the studies, none of the investigated models was fully explaining the data.^73^ In addition, experiments such as pump-push photocurrent spectroscopy provided evidence for the existence of traps as well as information about their kinetics.^74^ While there are examples of the ABC model appropriately describing experimental data especially at higher fluences,^75^ this is not the case generally, in particular when considering lower-fluence data where trap-related processes occur and the *A* parameter is too simple to capture the true behavior. Within the logic of the ABC model, radiative recombination is *Bn*,^2^ while it should be *Bnp* (always neglecting equilibrium concentrations), which is obviously only true for *n* = *p*. Given the ABC model hardcodes the condition that electron and hole densities are equal, a more complicated model was needed to include phenomena such as photodoping. A logical and minor modification of the ABC model would be to treat electron and hole densities as separate quantities and to replace the parameter *A* with the complete SRH equation. Furthermore, it would be necessary to invoke charge neutrality, i.e., to consider that a film would have to be charge-neutral and that negatively or positively charged trap states would have to be considered for the charge-neutrality condition.^10^ All these modifications keep the whole model analytically treatable, but they add additional parameters. The parameter *A* representing trap-assisted recombination translates into four separate parameters in the SRH model (electron and hole capture coefficients, trap density and trap energy). This step from one to four parameters was frequently considered to be adding too many additional degrees of freedom that may not reflect the information content of the experimental data. Thus, a peculiar compromise of adding three parameters (electron and hole capture and trap density) but omitting the trap depth became popular.^76,77^ Interestingly, a similarly truncated version of SRH was also used in the density functional theory community to calculate total capture coefficients from electron and hole capture.^78^ Recently, Wang et al.^79^ showed that the omission of detrapping (e.g., electron emission from a trap back to the conduction band) can lead to orders of magnitude errors (8 orders in the example shown in Figure 2 of ref 79) in the calculated total recombination rates for amphoteric traps, i.e., those that can have more than two charge states (+/0/−). This is primarily due to the problem that in the case of amphoteric traps without detrapping, one channel that has a low capture coefficient for either electrons or holes could completely fill and thereby clog the overall recombination channel. Experimentally, similar problems have arisen to explain the combination of power law decays (PL intensity scaling with 1/time^2^ at late times in transient PL data resulting from bimolecular recombination) and much lower-than-unity PL quantum efficiencies. This combination of features can be described quite straightforwardly using shallow traps^66^ that have a non-negligible rate of detrapping, but it cannot be described within the logic of the previously mentioned truncated SRH model. Figure 1 presents an overview of the evolution of recombination models for semiconductors and perovskites, including features that need to be considered for future developments. Table 1 summarizes how experiments could be used to identify features and parameters of different physical models.

**Figure 1:**
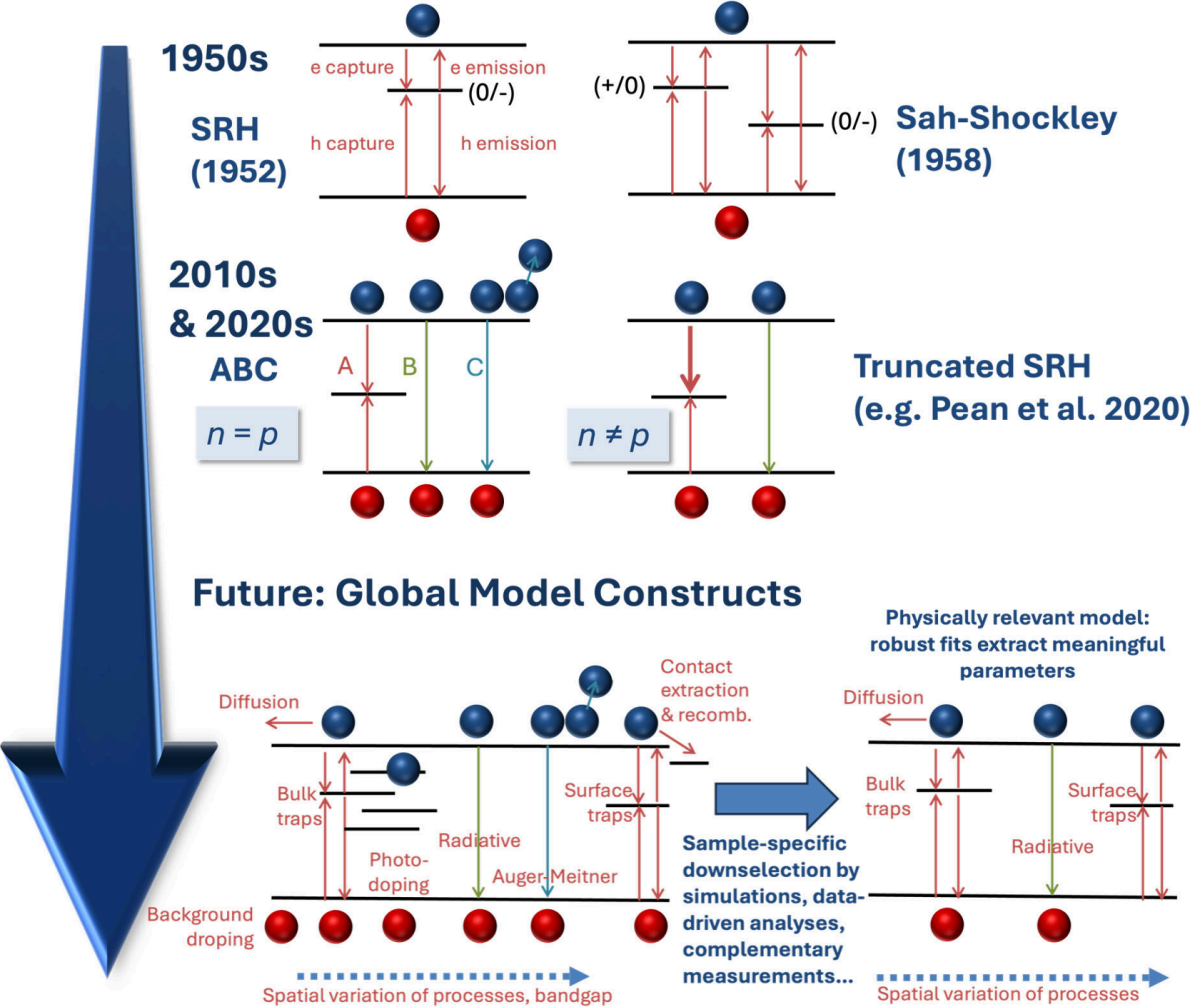
Evolution of models describing recombination in semiconductors in general (1950s) and metal halide perovskites in particular (2010s onward) and anticipated future developments. Note that the Shockley–Read–Hall (SRH)^80,81^ model was designed for singly charged defects (e.g., acceptor like (0/-) or donor-like (+/0)) defects. The Sah-Shockley^82^ model was an extension of SRH meant for amphoteric defects. In most work on recombination of inorganic solar cells, those two mechanisms would be accompanied by radiative and Auger recombination depending on the situation. The key feature of the ABC model is its hardcoding of the *n* = *p* condition that is needed to arrive at a single particle picture of recombination.

**Table 1:** Strategies to Increase the Discriminatory Power of Experiments to Identify Suitable Models and Explanations on How and Why They Work

strategy	why it works	refs
Transient experiment at different fluences	Covers a larger range in carrier densities. Can discriminate better between, e.g., shallow and deep traps.	^63,83^
High dynamic range transient experiments	Same as above.	^66,84^
Combination of tr-PL and transient absorption or transient photoconductivity	Sensitivity to *np* and *n* + *p*. Notices asymmetries in *n* vs *p* and is thereby sensitive to photodoping.	^68,85^
Tr-PL + steady state PL (PLQY)	Sensitive to, e.g., trap depth (via tr-PL slope) and photodoping (via slope of PLQY vs excitation flux). Combinations on half-cells could characterize interface recombination.^22,23^ Addition of initial tr-PL intensity vs fluence^40^ indicates doping level and further refines model parameters.	^66,73^
Pulse burst experiments in tr-PL (Hedgehog plots)	Fills up traps with several laser pulses in quick succession. Sensitive to trap density and trap filling parameters.	^86^
Horse plots (variation of repetition rate and fluence in tr-PL)	Sensitive to photodoping (via the slope of the neck of the horse) and to different recombination mechanisms (defects, radiative, Auger).	^73^

## Data Fitting

In addition to the question of what model to use to describe recombination, a separate question arose as to how to fit data originating from transient measurements. Initially, one might think that those questions are closely linked to each other. The choice of model should immediately dictate the type of function used to fit the data. For instance, if the recombination rate was thought to be predominantly linear in electron density, the electron density should decay exponentially as this is the solution of the associated differential equation governing the decay. The challenge here was that even for the seemingly simple ABC model, there was no simple analytical solution for the time-dependent carrier density. As in many practical situations, the ABC model was rather an AB model with at best a hint of Auger-Meitner recombination being visible at higher fluences or laser powers, a conceivable solution would have been to take the analytical solution for the time-dependent luminescence in the AB model (which is known and reasonably simple^87^) and use that to fit the data. However, this rather logical approach was very rarely pursued.^88^

Instead, three completely disjunct strategies were followed by the community. The majority of researchers were using a multiexponential approach to fit the transient PL or absorption data. This had the clear advantage of being easy to implement and to be numerically rather stable. In addition, it often provided decent results when visualized on semilogarithmic plots (linear time axes) and would only show reason for concern when looked at on a double-logarithmic scale.^84^ The key downside was that multiexponential fitting was completely physically and mathematically inconsistent with even the simplest conceivable recombination model (AB). Thus, the connection of the fitted decay times (usually a biexponential decay was used thereby producing two decay times and one constant offset for the noise level) to parameters of a model (AB­(C) or SRH) was not at all straightforward and therefore rarely attempted. The most abundantly used approach is to use a weighted mean of the two decay times of a biexponential decay as an effective lifetime, with this approach again lacking physical meaning. Another key downside of this approach is that a fast initial decay is implicitly considered to be indicative of poor material quality when the weighted mean approach is used. However, this is not necessarily the case when a fast and a slow time constant are averaged. Take the situation where the initial decay was dominated by radiative recombination, then a fast lifetime would be a good thing. A high radiative recombination coefficient means that the absorption coefficient is high and would therefore be good for a given photovoltaic material. Alternatively, a fast initial decay may originate from a fast electron or hole capture by a shallow defect. This would not be particularly relevant for the overall photovoltaic quality as, in this case, only the slower transition matters for recombination. Thus, the biexponential fit is not only decoupled from physical models and does not fit the data well, but it may also lead to a metric that is not directly correlated with the speed of recombination as it is intended and implied.

An alternative approach – and in many ways the ideal approach – was based on fitting a complete rate equation model to the experimental data. Especially for films, this was a perfectly logical approach that was moderately easy to implement and directly provided the parameters of a physically meaningful model. The key downside of this approach was the challenge of quantifying confidence in the result and the uniqueness of the fit. A possible solution that was explored in a small number of more recent studies was the combination of using a rate equation model with a Bayesian inference framework, where the likelihood of the parameters explaining the data is explicitly calculated and parameter correlations are visualized (see further discussion later).^76,89,90^ Table 2 provides a list of code that is freely available online that serves to fit transient data (mostly tr-PL) to a physical recombination model. Detailed descriptions of each of the codes are available in the publications cited and/or in the links provided.

**Table 2:** Tools and Scripts to Fit Data from Transient Experiments to a Physical Model

description of tool or script	link
Bayesian optimization combined with rate equation models for tr-PL or other transient experiments (also steady state experiments). Partly based on ref 76.	https://openpv-lab.github.io/optimPV/
Fitting tr-PL with Bayesian Inference. MCMC used for sampling the parameter space. Based on ref 90.	https://github.com/manuelkoberczerny/assessing-TRPL-with-bayesian-inference_and-MCMC
Fitting tr-PL with Bayesian Inference. Metropolis Hastings used for sampling the parameter space. Based on ref 100.	https://github.com/HagesLab/MetroTRPL
Fitting tr-PL and steady state PL simultaneously with (up to) three traps. Based on ref 66.	https://zenodo.org/records/10101259
Fitting tr-PL spectra to determine mobilities and lifetimes. Based on a combination of diffusion and recombination (no drift). Described in ref 99.	https://zenodo.org/records/14650223

Occasionally, a third approach to data fitting has been pursued^91^ based on the idea that macroscopic tr-PL measurements average over a certain lateral extension of the sample. If the sample is a polycrystalline thin film, it is fair to assume and has been corroborated experimentally in microscopic measurements,^92–94^ that sample quality and therefore also decay times vary spatially (laterally). A relatively simple function that can be interpreted as a distribution of exponential decay times is the so-called stretched exponential (exp­(−(*t*/τ)^β^), where the argument of the exponent (excluding the minus) is taken to the power of a parameter β that correlates with the width of the distribution. Lower values of β (approaching zero) imply broader distributions of decay times τ, while β = 1 recovers the exponential decay with a single decay time. The advantage of the approach is its simplicity in dealing with an actual issue of macroscopic measurements (namely lateral disorder in decay times) that may lead to locally perfectly exponential decays that translate into nonexponential decays after lateral averaging. The downside is the clear limitation to first order processes, meaning that we must start with locally exponential decays. Thus, in the nomenclature of the ABC model, the stretched exponential fit is a pure A model with disorder, which limits its use to situations where deep defects are dominating the decay. Another potential downside is that processes that might locally be nonexponential (i.e., radiative or shallow traps) could be misinterpreted (in macroscopic tr-PL measurements) as signatures of disorder as the additional degree of freedom in the stretched exponential enables better fits for curves that do not look purely exponential.

An intermediate step between plotting the raw data (usually PL vs time) and fitting the data was introduced in refs 19,95 and was based on the use of differential decay times plotted first as a function of time^19^ and then later as a function of carrier density or Fermi-level splitting.^95^ This approach was inspired by the way lifetime data is analyzed in the silicon solar cell community,^96^ which has used quasi-steady-state photoconductivity decay (QSSPC) data as a standard method since the mid-1990s^97^ for the purpose of inferring recombination parameters. The standard approach has been to plot the lifetime as a function of excess carrier density to disentangle low-level injection, high-level injection, and the Auger-Meitner limited region (see, e.g., Figures 4 and 5 in ref 98). In the perovskite community, this approach had to be slightly adapted given the absence of a well-defined minority carrier and the low doping densities. Here, data was nearly always in high-level injection (injected charge carrier density > ionized doping density at 0 V in the dark), but the decay times were still changing significantly with carrier density.^84^ A logical approach was therefore to use the geometric mean of carrier densities or the Fermi-level splitting as the *x*-axis for the decay times as both would result directly from the absolute PL intensity and the initial carrier concentrations that could be deduced from a measurement of the pulse energy per area.^95^ A second challenge is to calculate derivatives from noisy experimental data. This can often only be done by first fitting the experimental data with physically meaningless functions containing a sufficient number of parameters (choices for fit functions could be high order polynomials or rational functions, i.e., ratios of high order polynomials), thereby achieving a good fit, and then taking the derivative of these functions. Alternatively, smoothing of the data and binning (to reduce the number of different time points) can help to calculate meaningful derivatives. This approach has some key advantages relative to just studying the PL intensity vs delay time. First, in case of data that eventually becomes exponential at longer times, it allows one to directly read out the characteristic time of this exponential decay from the differential decay time^95^ and removes a strong need to fit the data with a model. Further, it allows us to directly see whether the data is nonexponential and shows the signatures of, e.g., shallow traps without the need to commit to a specific physical model to fit the data to. Finally, it allows us to combine measurements performed at different fluences in one plot^66,83^ and immediately highlights those regions that are affected by trapping and diffusion at early times^99^ and those regions that only depend on the average carrier density and are dominated by recombination and a quasi-equilibrium occupation of the trap states. The latter regions are the ones where the different fluences would overlap when plotted vs Fermi-level splitting or geometric carrier density mean. This distinction can be observed for instance in Figure 2E in ref 99.

## Challenges and Opportunities: Toward a Global Model

There has been promising progress over the years to develop recombination models based on known physical processes that best reproduce experimental data. A thorough understanding of recombination processes will allow identification of, for instance, the nature and abundance of particular carrier traps leading to performance losses. This will in turn allow more informed approaches to improve device efficiency. Furthermore, there are increasing links between carrier traps and instabilities; a notable example is the presence of hexagonal polytype impurities in formamidinium-rich absorber materials that act as deep traps for photoexcited holes^101^ and the very act of this hole trapping drives chemical reactions resulting in photoinstabilities.^102^ Thus, it is expected that complete recombination models will also guide efforts to further stabilize perovskite solar cells.

Nevertheless, there is currently no generally accepted model that perfectly captures all experimental behavior. This motivates an important challenge – defining a template of a global model that can be generally applied to halide perovskite absorbers that contains all key processes, and the set(s) of measurements to probe these processes. This model template can then be used to find the simplest model that describes a given sample with sufficient accuracy. Such a global model (template) must work on two levels: a practical approach that can be routinely and robustly used by device makers who require a means to quantitively assess alterations to absorber and device layers, and a second level which is a sufficiently (but not over-) complex model containing all processes to extract all relevant physical parameters. Once such a template of a model containing for instance *n* traps is developed, one specific implementation of a model following that template could be selected for a given sample that provides a good compromise between complexity and accuracy and that for instance contains 2 traps because those are needed to describe the observed features with sufficient accuracy. This approach aims to avoid a situation in which models become ever more complex with more fitting parameters that can fit to any data sets with multiple possible solutions (von Neumann’s famous “fitting an elephant”),^103,104^ whereby such extracted overfitted parameters then become meaningless. Occam’s Razor tells us a model should be as simple as it can be to describe the data and physical phenomena, and this should remain at the forefront of ongoing developments. In contrast to the danger of using too many parameters, we also want to mention the recent work of Heester et al.^105^ that shows how surprisingly restrictive certain parameters (such as the band offset) can be for the purpose of achieving a good fit of a model to rather simple experimental data (current–voltage curves in the example of Heester et al.). This finding therefore motivates the search for pairs of parameters and experiments where the information obtained from the experiment is particularly powerful at restricting the values of certain parameters.

Work to date tells us that any global model template would need to consider a number of physical processes that charge carriers undertake. In a neat absorber film, this includes, but is not limited to, radiative recombination, all processes included within the overarching framework of SRH including trapping and detrapping processes in either/both shallow and deep trap states (and, in general, a continuous distribution of states through the bandgap)^106^ as well as recombination, photodoping (i.e., the combination of SRH with the charge neutrality condition including the trap density), background doping, and Auger–Meitner processes – with individual densities of electrons and holes being independently tracked through each process. This approach would also need to account for the spatial distribution of the processes through the film – including surface recombination velocity and distinguishing of traps on the surface and bulk and carrier diffusion through the thickness of the film. In microscopic viewpoints, many of these processes will also vary laterally as the trap distributions likely vary on multiple length scales.^107^ When contacts are added to construct half or full devices, and bias applied, carrier extraction and other recombination processes (including additional traps related to contacts) will occur at the contacts. There may be additionally electric fields across the device stacks, in which case carrier drift (in addition to diffusion) will need to be considered, as well as movement of ions that will further mediate recombination processes and instabilities.^108^ These “charge collecting” scenarios provide further means to decouple the electron and hole populations through selective extraction of one/both, which in the end also impact other processes such as trapping and related photodoping that will be sensitive to asymmetries in electron and hole populations.^109^ This situation leads to a complicated, interrelated system that needs care in mathematical and physical construction. In the steady state, we can calculate the SRH or Sah-Shockley occupation functions and then multiply them with an arbitrary density of localized states in the band gap.^106^ Thus, it would be straightforward to arrive at a manageable model even with an arbitrarily complicated distribution of defects and use that to fit the data. However, in the transient case, this becomes a more complex challenge especially if a high number (or continuous distribution) of localized states have to be included, leading to a highly underdetermined system with many more unknowns than characteristic features in the data^110^ (see Table 1 for some experimental approaches to this). A simple example may be considering the excitation fluence under consideration – in a low fluence regime, Auger–Meitner processes are negligible and thus may be ignored. Another example may be examination of the shape of an experimental curve (such as tr-PL decay curve), together with simulations exploring changes to different terms, to isolate dominant processes – for instance whether one, two, or more trap energies are required. Such explorations of simulations and experiments will also allow qualitative explanation of different recombination regimes (such as exponential or power-law type, or transition between them). A further example might be isolating the diffusion component through use of spectrally resolved tr-PL measurements, allowing the remaining processes to be modeled without diffusion.^38,99,111^ Smart, dynamic coupling between simulation exploration and model construction will likely lead to fruitful avenues.^94^ This physics-informed approach could be conducted iteratively with data-driven algorithms to identify the simplest partial differential equations (and therefore model) that describe the data reasonably.^112^

There is hope in the fact that the contribution of each process will not be equal, and one could pinpoint the dominant processes to simplify the problem for the given sample to best focus the modeling and fitting procedures. The challenge is how to efficiently work out which processes are most relevant in a given sample to remove terms that provide negligible contributions.

Another key question is which combinations of measurements are leading to useful data that can be modeled and help infer material parameters. This question is closely linked to the question of what model is most appropriate to describe the physical reality of the experiments. If we take the ABC model as a convenient example, then we note that due to the ABC model only considering one concentration (electrons and holes being equal in density), the steady state and the transient solution of ABC provide the exact same information content. Whether you know how the recombination rate depends on carrier density (steady state) or how the carrier density after a pulse depends on time can be mathematically proven to be identical if the two experiments cover the same range of carrier densities. In realistic models, the electron and hole densities are separate and thus the densities of trapped charge carriers are explicitly considered. In these cases, transient and steady state measurements provide different information. That is, they cover different trajectories of the phase space of possible combinations of electron density, hole density and trapped charge densities. For instance, in a transient experiment, one may access the situation where the electron and hole densities are both high (directly after the pulse), while a trap might be nearly empty (as in the dark) and then see the trap being filled. This situation would never occur in steady state as the trap filling would always be in some equilibrium with the two bands governed by the steady state solution of SRH statistics. This distinction implies that steady-state and transient PL can actually provide highly complementary information that one, however, is only able to appreciate, access and use if one abandons models such as ABC that do not explicitly consider trapped charge densities.

Another aspect of inferring parameters from models is the dynamic range of the experiment. Even in the example of ABC, determining all three parameters from a hypothetical data set consistent with ABC would only be possible if that data set covers a sufficiently large range of carrier densities to observe the linear, quadratic and cubic regime of ABC. The dynamic range of carrier densities is limited by experimental sensitivity. For example, when using time-resolved PL from typical time-correlated single photon counting (TCSPC) setups achieving a high dynamic range is more challenging and is often only achieved by combining measurements with different fluences.^83,113^ Use of more sensitive setups such as intensified charge-coupled device (iCCDs) provide much better dynamic range for full decays, up to 7–9 orders of magnitude assuming high initial fluences, to allow sensitive probing of even weak, long-time processes.^66^ TCSPC, on the other hand, could be best used to understand and quantify trap filling, such as through hedgehog plots (cf. Table 1).^86^ Combinations of both approaches could make for fruitful avenues.

The probing of only a subset of the phase space has some important ramifications. First, no measurement could probe the full parameter space. Second, it is possible that an experiment can be conducted and suitably modeled to capture the key physical processes leading to the measured data, but this could entirely miss a key process (outside the accessed measurement space) relevant to a device such as a trap that would otherwise effectively be invisible to the measurement and analysis conducted. This latter problem motivates complementary measurement techniques to access a wider range of the phase space. One example would be the combined use of steady-state and time-resolved photoluminescence measurements, which access independent regions of the phase space. To illustrate this, just imagine the situation in tr-PL, where free electrons and holes are created near instantaneously with a laser pulse, whereas the trap occupation is initially the same as in the dark. In a steady-state experiment, this situation would never happen as the occupation of the trap is always set by the balance of capture and emission processes that leads to a time-independent trap occupation. Other complementary examples could be use of optical or electrical measurements such as transient surface photovoltage (to probe carriers building up and recombination in contacts),^114^ transient absorption^59^ or microwave conductivity^85,115^ (to probe processes proportional to the sum of electron and hole populations, rather than the product as measured in PL) or transient photoemission (to probe carriers in traps).^101^ Furthermore, there are still open research questions as to what the precise chemical nature^116^ is of the various traps (deep or shallow, including those contributing to second-order nonradiative), and further developments allowing corroborative measurements and calculations of trap nature, density, and depth would further boost correct modeling approaches.

**Table 3:** Guide on How To Analyze and Interpret Transient Data, Including Detailed Literature References for Further Reading^a^

process step	what to do
Measurement	Cover wide range of carrier densities using different fluences (see, e.g., ref 83) or high-dynamic-range-compatible setups (see, e.g., ref 95). For comparison on the same sample, see ref 66, Figure 2).
	Check for effect of repetition rate.^84,117^
	Measure background separately.^84^
Preprocessing	Find zero of time axis (usually, the end of the laser pulse).
	Background subtraction (see, e.g., ref 84 SI, page 21).
Calculate relevant decay time metrics	Fit data with sufficiently complex function (e.g., high order polynomial or rational function, see ref 95 page 15, or ref 83 SI, page 29).
	Calculate derivative from the fit (see, e.g., ref 95 page 3).
	Plot vs Fermi-level splitting for fitting/analysis (see ref 95 SI, page 24).
Visual inspection of data	Compare shape with typical simulated behavior of different recombination mechanisms (e.g., shallow trap vs deep trap; see, e.g., ref 118, Figures 6 and 9).
	Identify most likely recombination model.
Fit with recombination model	Fit decay time to appropriate recombination model (often rate equation model, see, e.g., ref 118, chapter 5)
	Infer parameters (see, e.g., the methods described in Table[Table tbl2]).
Comparison with other experimental data	Do global fits of one model to several data sets (e.g., steady-state PL and tr-PL; example is ref 66).
	Do simultaneous measurements (e.g., tr-PL and time-resolved photoconductivity, see ref 85).
	Infer parameters and quantify confidence (again, see the methods described in Table[Table tbl2]).

a Especially, the more technical details are sometimes difficult to find, therefore, specific references to the supporting information pages are made where we deemed it necessary.

Indeed, being able to reproduce experimental data using a physically robust model is one thing, but whether this provides meaningfully useful information (e.g., about the material or a device) is another. This comes down to what one might want from such analyses – a device maker may be most interested in the impact of a passivation agent (first ‘level’ of the global model), but a spectroscopist may be interested in ascertaining all physical parameters for a holistic understanding of the material (second ‘level’). The former may only require a subset of measurements compared to the latter, but it is important that the measurements cover the regime able to access the relevant parameters in each case. There are also open research questions as to how much influence a term such as a shallow trap, that may be important to describe tr-PL kinetics, will actually have on an electrical measurement and therefore whether drift-diffusion modeling to describe the latter needs to incorporate such processes. Identifying the key measurements, and regimes over which to measure, will be critical toward developing unified approaches.

Combinations of simulation software to define the model and development of smart data-driven fitting algorithms drawing on machine-learning algorithms with physics-informed models are likely to lead to success.

Once the model containing only the seemingly important parameters are included, a final challenge is robustly fitting the data using the model. Even fitting simulated data can have pitfalls and the step from fitting simulated data to fitting real data is far from trivial because experimental noise makes convergence of numerical fitting and the landscape of parameter exploration more complicated. Many fitting procedures will depend sensitively on initial parameters, meaning they will only explore a limited range for some parameters or will converge to local rather than global minima. This convergence (or not) will depend on the landscape of local slopes of the fitting algorithms and also the quality and even numerical form of the data – with convergence within shallow, slow-varying landscapes the most difficult. One could define reasonable physically meaningfully starting parameters based on analytical approximations to the numerical solutions (many analytical approximations to steady-state and transient PL are discussed in refs 87,118 ) and iterate accordingly, though careful exploration to ensure that the finalized parameters are independent of the starting parameters or fitting approach is crucial. Combinations of simulation software to define the model and development of smart data-driven fitting algorithms drawing on machine-learning algorithms (see Table 2) with physics-informed models are likely to lead to success. We summarize in Table 3 a guide on how to approach measurement, analysis and interpretation of transient data.

There is enormous opportunity for halide perovskite semiconductors to play a key role in decarbonisation over the coming decades, in particular through high-performance photovoltaics. However, there is an urgent need to ensure all recombination processes are understood to drive further technological developments and to develop robust, quantitative methods to assess these processes that will underpin widespread manufacturing. We expect this perspective will provide useful guidelines and directions toward reaching these ambitions and will be applicable for further development of other emerging semiconductors.
